# Generation of Viable Candida albicans Mutants Lacking the “Essential” Protein Kinase Snf1 by Inducible Gene Deletion

**DOI:** 10.1128/mSphere.00805-20

**Published:** 2020-08-19

**Authors:** Austin Mottola, Sonja Schwanfelder, Joachim Morschhäuser

**Affiliations:** a Institut für Molekulare Infektionsbiologie, Universität Würzburg, Würzburg, Germany; University of Georgia

**Keywords:** *Candida albicans*, Snf1, conditional mutants, essential genes, protein kinases

## Abstract

The protein kinase Snf1, a member of the highly conserved AMP-activated protein kinase family, is a central regulator of metabolic adaptation. In the pathogenic yeast Candida albicans, Snf1 is considered to be essential, as previous attempts by different research groups to generate homozygous *snf1*Δ mutants were unsuccessful. We aimed to elucidate why Snf1 is required for viability in C. albicans by generating *snf1*Δ null mutants through forced, inducible gene deletion and observing the terminal phenotype before cell death. Unexpectedly, we found that *snf1*Δ mutants were viable and could grow, albeit very slowly, on rich media containing the preferred carbon source glucose. Growth was improved when the cells were incubated at 37°C instead of 30°C, and this phenotype enabled us to isolate homozygous *snf1*Δ mutants also by conventional, sequential deletion of both *SNF1* alleles in a wild-type C. albicans strain. All *snf1*Δ mutants could grow slowly on glucose but were unable to utilize alternative carbon sources. Our results show that, under optimal conditions, C. albicans can live and grow without Snf1. Furthermore, they demonstrate that inducible gene deletion is a powerful method for assessing gene essentiality in C. albicans.

**IMPORTANCE** Essential genes are those that are indispensable for the viability and growth of an organism. Previous studies indicated that the protein kinase Snf1, a central regulator of metabolic adaptation, is essential in the pathogenic yeast Candida albicans, because no homozygous *snf1* deletion mutants of C. albicans wild-type strains could be obtained by standard approaches. In order to investigate the lethal consequences of *SNF1* deletion, we generated conditional mutants in which *SNF1* could be deleted by forced, inducible excision from the genome. Unexpectedly, we found that *snf1* null mutants were viable and could grow slowly under optimal conditions. The growth phenotypes of the *snf1*Δ mutants explain why such mutants were not recovered in previous attempts. Our study demonstrates that inducible gene deletion is a powerful method for assessing gene essentiality in C. albicans.

## Abstract (precis)

Essential genes are those that are indispensable for the viability and growth of an organism. Previous studies indicated that the protein kinase Snf1, a central regulator of metabolic adaptation, is essential in the pathogenic yeast Candida albicans, because no homozygous *snf1* deletion mutants of C. albicans wild-type strains could be obtained by standard approaches. In order to investigate the lethal consequences of *SNF1* deletion, we generated conditional mutants in which *SNF1* could be deleted by forced, inducible excision from the genome. Unexpectedly, we found that *snf1* null mutants were viable and could grow slowly under optimal conditions. The growth phenotypes of the *snf1*Δ mutants explain why such mutants were not recovered in previous attempts. Our study demonstrates that inducible gene deletion is a powerful method for assessing gene essentiality in C. albicans.

## INTRODUCTION

Metabolic flexibility is important for the ability of the opportunistic pathogenic yeast Candida albicans to adapt to different environments during colonization and infection of its human host (1). The heterotrimeric protein kinase SNF1, a member of the highly conserved AMP-activated protein kinase family, plays a key role in the adaptation of C. albicans to glucose limitation and utilization of alternative carbon sources (2). In C. albicans, the SNF1 complex consists of the catalytic α-subunit Snf1, the regulatory γ-subunit Snf4, and one of the two β-subunits Kis1 and Kis2 (3). The γ-subunit Snf4 is required to relieve the N-terminal catalytic domain of Snf1 from autoinhibition by the C-terminal regulatory domain, and the β-subunits Kis1 and Kis2 presumably mediate interactions with target proteins, in analogy to the function of their counterparts in the model yeast Saccharomyces cerevisiae (2, 4, 5). SNF1 functionality also requires phosphorylation of Snf1 at Thr208 in the activation loop by the upstream activating kinase Sak1 (2). Deletion mutants lacking the γ-subunit Snf4 cannot grow on carbon sources other than glucose, and mutants lacking the Snf1-activating kinase Sak1 have similar, albeit slightly milder phenotypes (2).

Surprisingly, the catalytic subunit Snf1 seems to be essential in C. albicans, which is not the case in S. cerevisiae, as numerous attempts by different research groups to construct homozygous *snf1*Δ mutants have failed. Petter et al. (6) used a recyclable *URA3* selection marker to delete *SNF1* in a uridine-auxotrophic derivative of the C. albicans wild-type strain SC5314 by allelic replacement. After deletion of one *SNF1* allele, various efforts to inactivate the second endogenous *SNF1* allele were unsuccessful, and all uridine-prototrophic transformants of the heterozygous mutant retained a functional *SNF1* copy (6). Enloe et al. used a *ura3-ARG4-ura3* cassette in an auxotrophic *ura3 arg4* parental strain to assess the essentiality of *SNF1* and other genes (7). When targeting nonessential genes with this approach, homozygous mutants can be obtained that contain the original *ura3-ARG4-ura3* cassette in one allele and a recombined functional *URA3* marker in the other allele. If a gene is essential, prototrophic transformants contain a duplicated third copy of the wild-type gene in addition to two inactivated copies. The latter was the case for *SNF1*, further supporting that Snf1 is essential for viability in C. albicans. More recently, Vyas et al. used CRISPR-Cas9 technology to generate *snf1* mutants (8). A transformant in which both *SNF1* alleles were placed under the control of a glucose-repressible promoter failed to grow under repressing conditions, in line with the predicted essentiality of Snf1. Interestingly, however, the wild-type *SNF1* alleles could be replaced by a “kinase-dead” allele encoding an enzymatically inactivated Snf1 protein. These mutants showed various phenotypic defects but grew well on glucose-containing rich medium, indicating that the kinase activity of Snf1 is not required for viability. In our laboratory, we used the *SAT1*-flipping strategy to inactivate *SNF1* in the wild-type strain SC5314 (2). Although either of the two *SNF1* alleles, which were distinguished by a restriction site polymorphism, could be deleted, no homozygous *snf1*Δ mutants were obtained after transformation of the heterozygous mutants with the same deletion cassette and selection for nourseothricin-resistant mutants on rich medium, as expected for an essential gene.

While these studies provided strong evidence for the essentiality of Snf1 in C. albicans, the reason for this remained unresolved (but see below). We therefore decided to generate *snf1*Δ mutants by inducible gene deletion (9) and observe the terminal phenotype of the null mutants before cell death. The conditional deletion mutants are generated as follows. After deletion of one allele of the target gene, a functional copy of the gene that is flanked by direct repeats of the recognition sequence of the site-specific recombinase FLP is inserted at an ectopic genomic locus in the heterozygous mutants. The second endogenous allele of the gene can then be deleted. Next, the *FLP* gene is integrated under the control of the tightly regulated, inducible *SAP2* promoter. After passage of the conditional mutants in *SAP2*-inducing medium, the deletable copy is excised by FLP-mediated recombination from the vast majority of the cells, generating an almost pure population of null mutants that can be phenotypically studied (9). This strategy provides firm proof for the essentiality (or not) of a gene, because true null mutants are obtained whose viability can be tested under any desired condition (as opposed to conditional mutants that have to be observed under specific, nonpermissive conditions).

## RESULTS

### Construction of inducible *snf1*Δ mutants.

**FIG 1:**
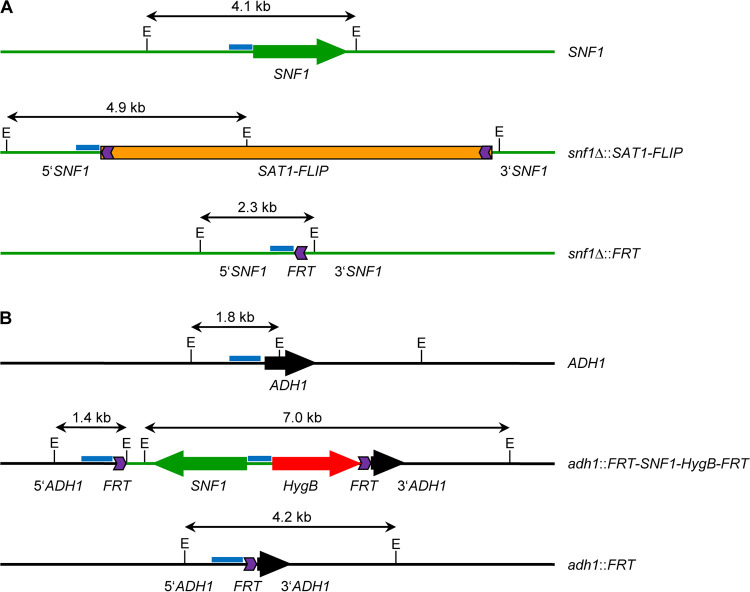
Schematic of the genomic *SNF1* and *ADH1* loci in the wild type and *snf1*Δ mutants. (A) *SNF1* locus in the parental strain SC5314 (top) and after integration (middle) and subsequent excision (bottom) of the *SAT1* flipper cassette. (B) *ADH1* locus in the parental strain SC5314 (top) and after integration (middle) and excision (bottom) of the deletable *SNF1* copy. The sizes of EcoRI fragments hybridizing with the indicated *SNF1*- and *ADH1*-specific probes (blue bars) are given. *SNF1* sequences are shown in green and *ADH1* sequences in black (the *SNF1* and *ADH1* coding sequences are represented by the green and black arrows); the *HygB* marker is illustrated by the red arrow and the *SAT1* flipper cassette by the orange bar; the lilac chevrons indicate the orientation of the 34-bp *FRT* sequence (not drawn to scale).

**FIG 2:**
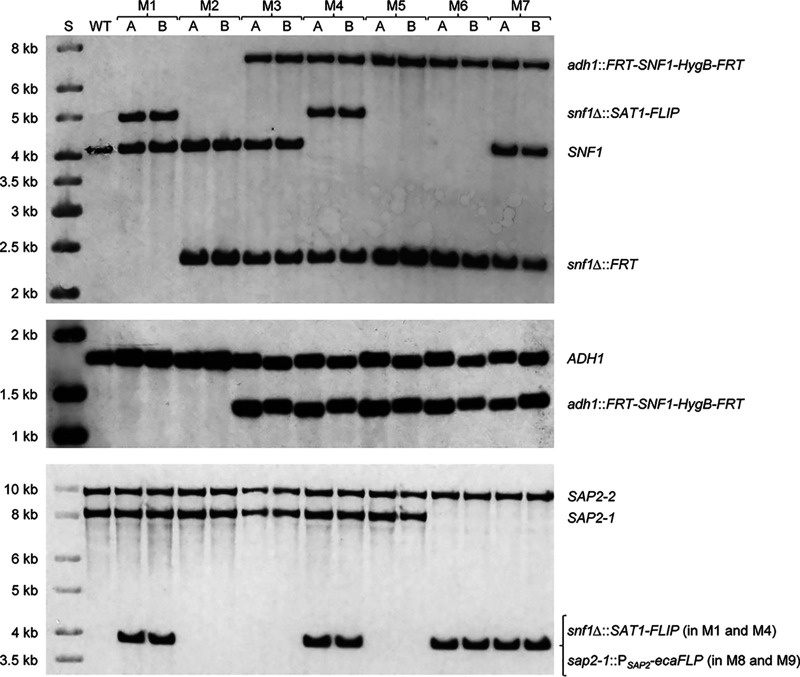
Construction of the conditional *snf1*Δ mutants and control strains. Genomic DNA of the wild-type strain SC5314 (WT) and the two independently generated A and B series of mutants (M1 to M7) was analyzed by Southern hybridization with a 5′ *SNF1* probe (top), a 5′ *ADH1* probe (middle), and a 5′ *SAP2* probe (bottom). The alleles represented by the hybridizing fragments are indicated on the right, and the sizes of a marker (S) are given on the left. The blots document the integration of the *SAT1*-flipper cassette into the first *SNF1* allele (M1), excision of the *SAT1* flipper cassette (M2), integration of the deletable *SNF1* copy into one of the *ADH1* alleles (M3), integration of the *SAT1*-flipper cassette into the second endogenous *SNF1* allele (M4), excision of the *SAT1* flipper cassette (M5), and integration of the *ecaFLP* gene under the control of the *SAP2* promoter into the *SAP2-1* allele of the conditional mutants (M6) and control strains (M7). The genomic DNA was digested with EcoRI (top and middle blots) or ClaI (bottom blot). The upstream region of *ADH1* allele 2 is slightly smaller than that of allele 1 due to several small deletions (recorded in assembly 19 of the SC5314 genome sequence in the *Candida* Genome Database), explaining the observed size differences.

To construct conditional *snf1*Δ mutants, we made some modifications to the strategy that was previously established for the generation of inducible *cdc42*Δ mutants (9). Instead of using the *URA3* flipper cassette for targeted gene replacement in the auxotrophic *ura3*Δ strain CAI4, the *SAT1* flipper cassette was used to delete the endogenous *SNF1* alleles in the prototrophic wild-type reference strain SC5314. An FLP-deletable *SNF1* copy was ectopically integrated at the *ADH1* instead of the *ACT1* locus, because derivatives of strain SC5314 that lack one *ADH1* allele did not exhibit a detectable growth or fitness defect in a previous study (10). The hygromycin resistance marker *HygB* (11) was used instead of the mycophenolic acid resistance marker *MPA*^R^ to select transformants containing the FLP-deletable *SNF1* copy, because use of the *MPA*^R^ marker requires long incubation on selection plates and results in a high frequency of undesired integration events (12). Finally, the *caSAT1* marker was used instead of the *URA3* marker to integrate the *ecaFLP* (enhanced *Candida*-adapted *FLP*) gene under the control of the *SAP2* promoter into the *SAP2-1* allele. Two independent series of inducible *snf1*Δ mutants were generated, starting from two previously constructed heterozygous mutants in which one or the other endogenous *SNF1* allele was deleted (2). In addition, we constructed two otherwise identical control strains that retain one of the endogenous *SNF1* alleles and should therefore still grow normally after excision of the FLP-deletable ectopic copy (just like the original heterozygous mutants from which they were derived). The construction of the mutants is illustrated and documented in Fig. 1 and 2.

### Generation of *snf1*Δ null mutants.

**FIG 3:**
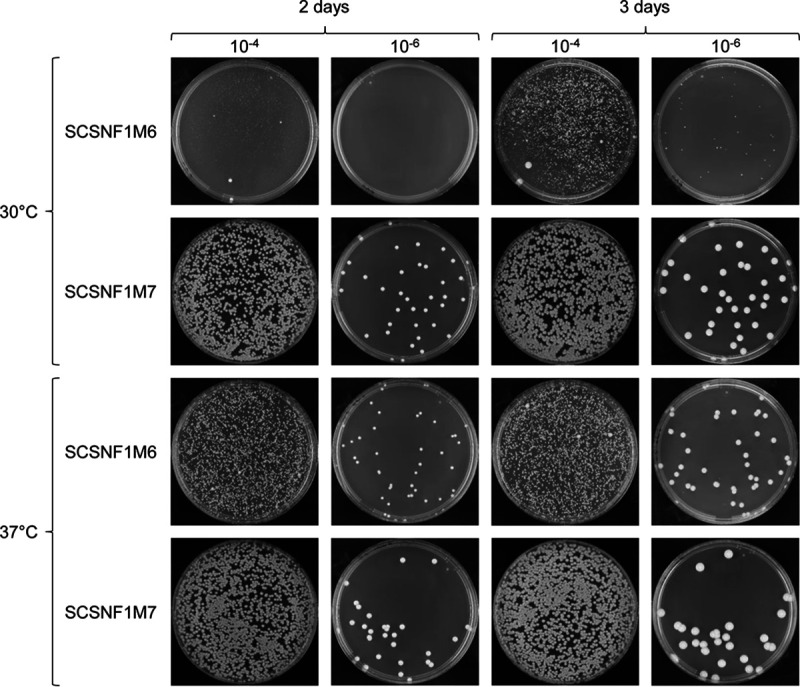
Plating efficiency of the conditional *snf1*Δ mutants SCSNF1M6A and -B and the control strains SCSNF1M7A and -B after FLP-mediated excision of the deletable *SNF1* copy. Overnight cultures of the strains in inducing medium YCB-BSA-YE were serially diluted, spread on YPD plates, and incubated at 30°C or 37°C. Plates were photographed after 2 and 3 days of growth. Note that corresponding plates from different days are not in exactly the same orientation. The two independently generated series of mutants gave the same result, and only one of them is shown in each case.

**FIG 4:**
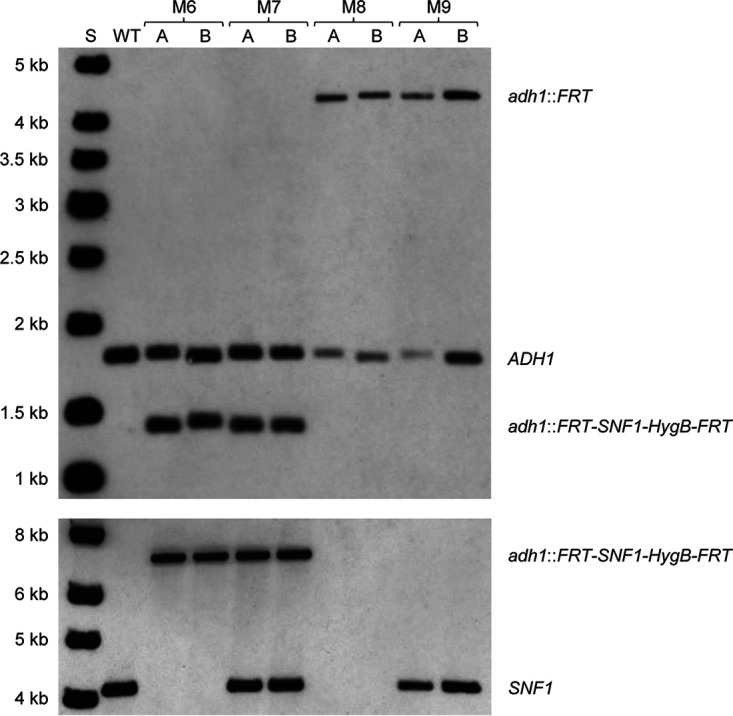
Genetic analysis of the *snf1*Δ mutants SCSNF1M8A and -B (M8) and the control strains SCSNF1M9A and -B (M9). EcoRI-digested genomic DNA of the strains was analyzed by Southern hybridization with a 5′ *ADH1* probe (top), to verify the induced genomic alteration at the *ADH1* locus, and with a probe from the *SNF1* coding sequence (bottom), to confirm the absence of *SNF1* in the null mutants. The alleles represented by the hybridizing fragments are indicated on the right, and the sizes of a marker (S) are given on the left. The wild-type strain SC5314 (WT) and the parental strains SCSNF1M6A and -B (M6) and SCSNF1M7A and -B (M7) are included for comparison.

The conditional *snf1*Δ mutants SCSNF1M6A and -B and the control strains SCSNF1M7A and -B (see Table S1 in the supplemental material) were inoculated into the *SAP2*-inducing medium YCB-BSA-YE (see Materials and Methods for definitions of media) and grown overnight to promote FLP-mediated excision of the deletable *SNF1* copy. The *SAP2-1* promoter is induced at the late stages of growth under these conditions (9, 13–15), and as expected from these previous studies, both the conditional mutants and the control strains had reached high densities in the overnight cultures. Serial dilutions of the cultures were plated on YPD plates and incubated for 2 days at 30°C to allow colony development. The frequency of normally growing colonies was reduced by about 3 orders of magnitude in the conditional mutants compared with the control strains, demonstrating that the inducible gene deletion had occurred with high efficiency (Fig. 3). However, the induced *snf1*Δ mutants produced tiny colonies that continued to grow after longer incubation of the plates, and the number of the colonies was comparable to those of the normally growing control strains. This unexpected result demonstrated that *snf1*Δ mutants are viable and can grow, albeit very slowly. Interestingly, the *snf1*Δ mutants grew much better when the plates were incubated at 37°C instead of 30°C (Fig. 3). We confirmed the deletion of *SNF1* in the slowly growing derivatives of SCSNF1M6A and -B by genetic analysis. Southern hybridization analysis of randomly picked clones with an *ADH1*-specific probe demonstrated the excision of the deletable *SNF1* copy in the induced derivatives of the conditional mutants and control strains, and hybridization with a probe from the *SNF1* coding sequence confirmed the absence of the gene in the *snf1*Δ mutants (Fig. 4, compare with the schematic in Fig. 1B). Two independent *snf1*Δ mutants (SCSNF1M8A and -B, derived from SCSNF1M6A and -B, respectively) and otherwise identical control strains (SCSNF1M9A and -B, derived from SCSNF1M7A and -B, respectively) were kept and used for phenotypic assays.

10.1128/mSphere.00805-20.1 TABLE S1 C. albicans strains used in this study. Download Table S1, XLSX file, 0.02 MB. Copyright © 2020 Mottola et al. 2020 Mottola et al. This content is distributed under the terms of the Creative Commons Attribution 4.0 International license.

### Phenotypic comparison of *snf1*Δ and *snf4*Δ mutants.

**FIG 5:**
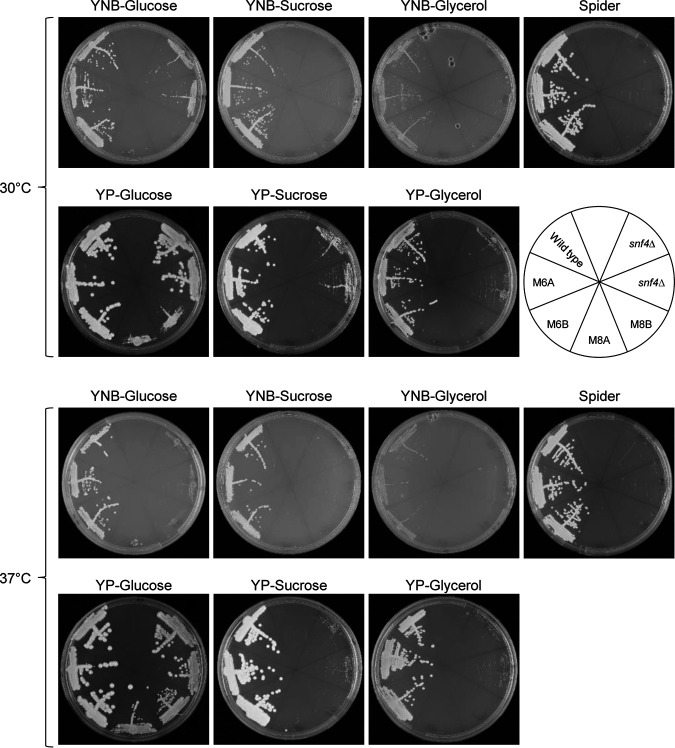
Growth of *snf1*Δ mutants and control strains on different carbon sources. YPD overnight cultures of the wild-type strain SC5314, the conditional *snf1*Δ mutants SCSNF1M6A and -B (M6), the *snf1*Δ null mutants SCSNF1M8A and -B (M8), and *snf4*Δ mutants (SCSNF4M4A and -B) were streaked on the indicated agar plates and grown for 2 days at 30°C or 37°C.

As explained in the introduction, C. albicans mutants lacking components of the SNF1 complex have growth defects on alternative carbon sources, the severity of which depends on the specific carbon source and the nature of the mutation. For example, *sak1*Δ mutants have only a mild growth defect on sucrose but a strong growth defect on glycerol, while *snf4*Δ mutants cannot grow on either sucrose or glycerol as the sole carbon source and even exhibit reduced growth on glucose (2). We therefore directly compared the growth of *snf1*Δ and *snf4*Δ mutants on glucose, sucrose, and glycerol. As can be seen in Fig. 5, the *snf1*Δ mutants grew much more poorly than the *snf4*Δ mutants on glucose, both on minimal medium (YNB-glucose), on which the *snf1*Δ mutants hardly grew at all, and on rich medium (YP-glucose). Growth differences between *snf1*Δ and *snf4*Δ mutants were also observed on YP-based media containing sucrose or glycerol, on which the *snf4*Δ mutants showed weak growth but the *snf1*Δ mutants could not grow. The parental conditional mutants SCSNF1M6A and -B grew as well as the wild type under all tested conditions, demonstrating that the single, deletable *SNF1* copy in these strains was sufficient for normal growth. Interestingly, the growth defect of the *snf4*Δ mutants was exacerbated at 37°C compared to 30°C, as opposed to the improved growth of the *snf1*Δ mutants on YPD plates at the higher temperature. This was especially evident on YNB-glucose plates, on which the *snf4*Δ mutants grew almost as poorly as the *snf1*Δ mutants at 37°C, and on YP-sucrose (Fig. 5). Therefore, the regulatory subunit Snf4 appears to be more critical for Snf1 function at the elevated temperature.

**FIG 6:**
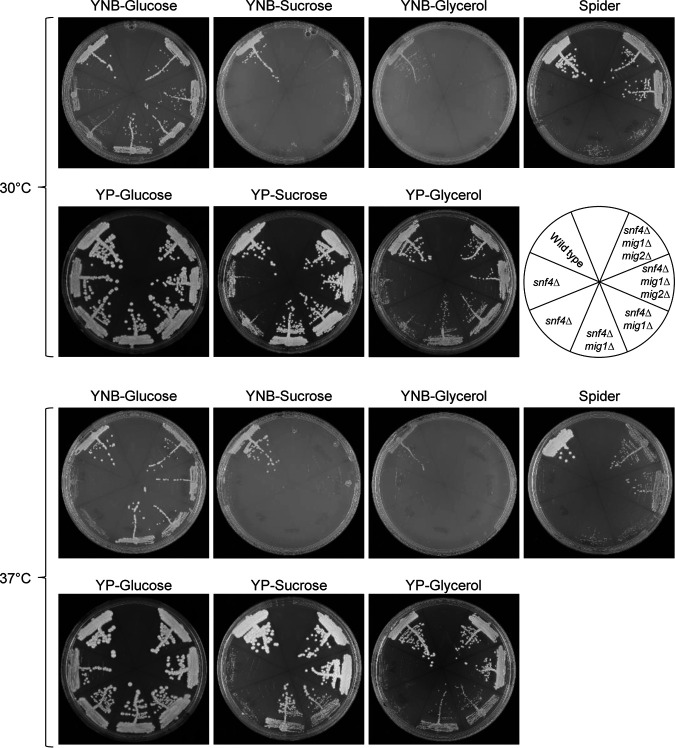
Deletion of *MIG1* and *MIG2* improves growth of *snf4*Δ mutants. YPD overnight cultures of the wild-type strain SC5314, *snf4*Δ single mutants (SCSNF4M4A and -B), *snf4*Δ *mig1*Δ double mutants (SCΔ*snf4*MIG1M4A and -B), and *snf4*Δ *mig1*Δ *mig2*Δ triple mutants (SCΔ*snf4*Δ*mig1*MIG2M4A and -B) were streaked on the indicated agar plates and grown for 2 days at 30°C or 37°C.

Recently, Lagree et al. reported that *SNF1* could be deleted in mutants lacking the repressor Mig1, indicating that unrestricted repression of Mig1 target genes in the absence of Snf1 explains the presumed essentiality of Snf1 (16). In our lab, we had also investigated the role of Mig1 in the regulatory network controlled by the SNF1 complex and deleted *MIG1* as well as both *MIG1* and the functionally related *MIG2* in our *snf4*Δ mutants. We found that the absence of these repressors partially alleviated the growth defects of the *snf4*Δ mutants (Fig. 6). Deletion of *MIG1* strongly improved the growth of the *snf4*Δ mutants on YP-sucrose and YP-glycerol, at both 30°C and 37°C, but was not sufficient to enable growth of the mutants on YNB-sucrose and YNB-glycerol. The additional deletion of *MIG2* further improved growth on YP-sucrose and YP-glycerol and also allowed some growth on YNB-sucrose. We also compared the growth of the mutants on Spider medium, which contains mannitol as carbon source and was used in the study by Lagree et al. (16) to investigate mutant phenotypes. Similar to what was observed with the other alternative carbon sources, the *snf1*Δ and *snf4*Δ mutants did not grow on Spider medium, and growth of the *snf4*Δ mutants was strongly improved when *MIG1* and *MIG2* were additionally deleted (Fig. 5 and 6). These results argued that the viability of our *snf1*Δ mutants was not caused by suppressor mutations in *MIG1* and/or *MIG2*, because the *snf1*Δ mutants could only grow slowly on YPD medium, but not on alternative carbon sources. Furthermore, sequencing of the *MIG1* and *MIG2* alleles of the *snf1*Δ mutants SCSNF1M8A and -B demonstrated that no mutations had occurred in the coding regions of these genes (see Materials and Methods for details).

### An enzymatically inactive Snf1 is nonfunctional.

**FIG 7:**
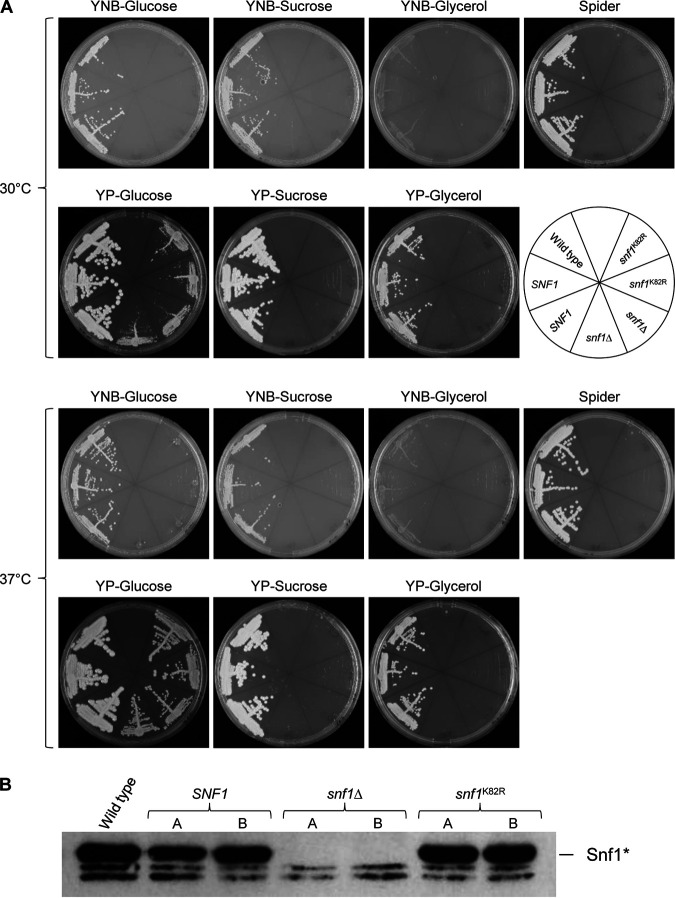
An enzymatically inactive Snf1 is nonfunctional. (A) YPD overnight cultures of the wild-type strain SC5314, strains SCSNF1M9A and -B retaining one of the endogenous *SNF1* alleles (*SNF1*), the homozygous mutants SCSNF1M8A and -B (*snf1*Δ), and strains SCSNF1M13A and -B containing the “kinase-dead” allele (*snf1*^K82R^) were streaked on the indicated agar plates and grown for 2 days at 30°C or 37°C. (B) Detection of phosphorylated Snf1 in the same strains. Protein extracts were prepared from cells grown to log phase in YPD medium at 37°C and analyzed by Western blotting with an antibody against Thr208-phosphorylated Snf1. The two weak, faster-migrating bands represent cross-reacting proteins.

A previous report suggested that the enzymatic activity of Snf1 is not required for the viability of C. albicans, because mutants could be obtained in which the wild-type *SNF1* alleles were replaced by a “kinase-dead” allele encoding a K81R mutation in the ATP-binding pocket of Snf1 (8). These mutants grew well on YPD plates at 37°C but exhibited growth defects at lower temperatures and could not grow on maltose as an alternative carbon source. We therefore compared our *snf1*Δ null mutants with strains that contained a single *snf1*^K82R^ allele (the two *SNF1* alleles in strain SC5314 differ by the length of a polyhistidine codon tract, and K81 mentioned above corresponds to K82 in the Snf1 reference sequence). To this aim, we generated strains that retained the *snf1*^K82R^ allele after FLP-mediated excision of the deletable *SNF1* copy (see Materials and Methods for details). The so-generated strains SCSNF1M13A and -B are otherwise isogenic with the *snf1*Δ null mutants SCSNF1M8A and -B and the control strains SCSNF1M9A and -B that retain a wild-type *SNF1* allele. We did not observe differences between the kinase-dead mutants and *snf1*Δ null mutants under any of the tested growth conditions (Fig. 7A). Western blot analysis demonstrated that the levels of Thr208-phosphorylated wild-type and mutant Snf1 proteins were comparable in strains containing a single copy of the corresponding allele (Fig. 7B). These results argue that an enzymatically inactive Snf1 is nonfunctional.

### Generation of “conventional” homozygous *snf1*Δ mutants.

**FIG 8:**
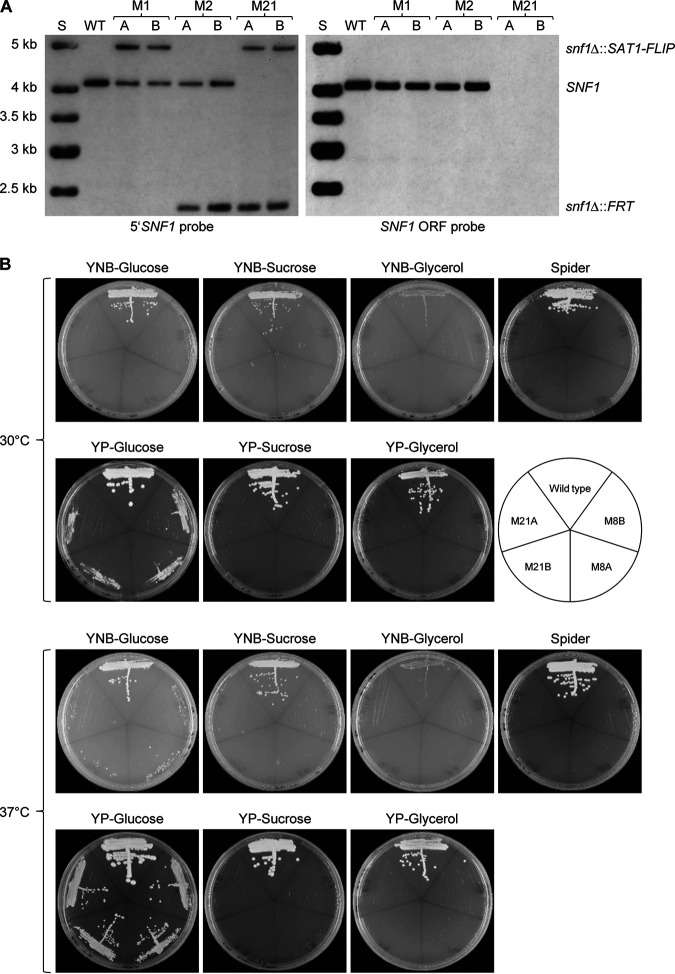
Phenotype of *snf1*Δ mutants generated by conventional gene deletion. (A) Confirmation of *SNF1* deletion in the *snf1*Δ mutants SCSNF1M21A and -B (M21) by Southern hybridization of EcoRI-digested genomic DNA of the strains with a 5′ *SNF1* probe and with a probe from the *SNF1* coding sequence. The alleles represented by the hybridizing fragments are indicated on the right, and the sizes of a marker (S) are given on the left of the blots. The wild-type strain SC5314 (WT) and the heterozygous mutants SCSNF1M1A and -B (M1) and SCSNF1M2A and -B (M2) are included for comparison. (B) YPD overnight cultures of the wild-type strain SC5314 and the *snf1*Δ mutants SCSNF1M8A and -B (M8) and SCSNF1M21A and -B (M21) were streaked on the indicated agar plates and grown for 2 days at 30°C or 37°C.

The poor growth of the *snf1*Δ mutants on YPD plates at 30°C explains why clones in which the remaining wild-type *SNF1* allele was replaced by the *SAT1* flipper cassette after the second round of transformation were not recovered in our previous study (2). Nourseothricin-resistant transformants are routinely isolated after 2 days of growth on the selection plates, because some spontaneously resistant, untransformed cells can also appear as small colonies after prolonged incubation (17, 18). Since *SNF1* was reported to be essential by several other groups (see introduction), we had made no further efforts to identify homozygous mutants in that previous study. On YNB-glucose plates, the *snf1*Δ mutants obtained by inducible gene deletion in our present work grew hardly at all (Fig. 5), which may explain the failure of other researchers to obtain homozygous *snf1*Δ mutants from auxotrophic host strains, which requires selection on appropriate minimal media (6, 7, 16). However, the conditional *snf1* mutant constructed by Vyas et al. was unable to grow after transfer to nonpermissive conditions, providing strong evidence that *SNF1* is essential (8). We therefore considered the possibility that the ectopically integrated, deletable *SNF1* copy was less functional than the endogenous alleles, which might have selected for suppressor mutations that allowed the survival and growth of the cells after the induced gene deletion. To bypass this potential caveat, and exploiting our finding that the *snf1*Δ mutants grew much better at 37°C than at 30°C, we transformed the heterozygous mutants SCSNF1M2A and -B again with the *SNF1* deletion cassette and incubated the selection plates for 3 days at 37°C. Besides normally growing transformants (which probably had integrated the *SAT1* flipper cassette into the already-inactivated *SNF1* allele or at an ectopic locus) and slowly growing untransformed cells that acquired spontaneous low-level nourseothricin resistance (see above), we recovered homozygous *snf1*Δ mutants in which the remaining endogenous *SNF1* allele was deleted and which grew as small colonies, as expected. We kept two independent homozygous mutants, generated from each of the two heterozygous parents, for phenotypic analysis (strains SCSNF1M21A and -B [Fig. 8A]). The homozygous *snf1*Δ mutants obtained directly after the gene replacement were used to avoid the additional subculturing required for marker recycling during which suppressor mutations might arise, especially in the induction medium YCB-BSA-YE, in which the *snf1*Δ mutants hardly grew at all. We then compared the phenotypes of the *snf1*Δ mutants generated by conventional gene deletion (SCSNF1M21A and -B) and the *snf1*Δ mutants obtained after induced gene deletion (SCSNF1M8A and -B). As can be seen in Fig. 8B, both types of *snf1*Δ mutants displayed the same slow growth on glucose and inability to grow on other carbon sources. This confirms that *snf1*Δ mutants are viable and can grow on rich media with glucose as carbon source, particularly at 37°C.

## DISCUSSION

The initial aim of this study was to elucidate why *SNF1* is an essential gene in C. albicans, by observing the terminal phenotype of null mutants obtained via forced gene deletion. The inducible gene deletion approach described in an earlier study (9) and modified here for use in wild-type C. albicans strains is a highly efficient way to achieve this goal, because it results in an almost pure population of null mutant cells that are all derived independently from a normally growing parental strain after a single passage in inducing medium. It can therefore provide definite proof of whether a gene is essential or not, because viability and growth of the null mutants can be tested under any desired condition. If the gene is indeed essential, the cells cannot grow, and cell viability/death can be assessed over time with appropriate methods. If the null mutants are viable and can grow, even if only under specific conditions, the gene is not essential, and this was unexpectedly found to be the case for *SNF1* in our present work. Essentiality depends on the genetic background, and one caveat is that suppressor mutations may allow growth of cells lacking a normally essential gene. This has recently been reported also for *SNF1*, which could be deleted in strains that lacked the repressor Mig1 (16). In principle, it is possible that our *snf1*Δ mutants had acquired such a suppressor mutation after the induced gene deletion to allow visible colony formation within 2 to 3 days. However, this would have to have occurred independently in every *snf1*Δ cell of the population, because the number of colonies was comparable in *snf1*Δ and control cells (Fig. 3), and such a high mutation rate appears very unlikely. A more realistic possibility is that a suppressor mutation might already have occurred in the parental conditional mutants if the deletable *SNF1* copy was less functional, thus fostering the acquisition of mutations that improved growth and enabled survival of the cells after subsequent deletion of *SNF1*. This would have to have happened in both independently generated conditional mutants after the deletion of the second endogenous *SNF1* allele in strains SCSNF1M4A/B and during the subsequent propagation steps that were necessary to obtain the final conditional mutants SCSNF1M6A/B. We therefore circumvented this potential problem by a renewed attempt to delete the remaining wild-type *SNF1* allele directly in the normally growing heterozygous mutants, taking advantage of the observation that the *snf1*Δ mutants obtained by induced gene deletion grew much better at 37°C than at 30°C. This enabled the successful isolation of homozygous *snf1*Δ mutants, which exhibited the same phenotypes as the *snf1*Δ mutants obtained by inducible gene deletion, i.e., slow growth on rich glucose-containing medium and inability to utilize alternative carbon sources. As already outlined above, these observations explain the previous failure by several research groups to generate *snf1*Δ mutants.

Yet, there is still one conundrum that is difficult to explain. The conditional *SNF1* knockdown mutant constructed by Vyas et al. (8), which expresses *SNF1* under the control of the *MAL2* promoter, failed to grow on YPD plates even at 37°C, which is in contrast to the phenotype of our *snf1*Δ mutants. This again may hint at the possibility that all our *snf1*Δ mutants contain suppressor mutations or epigenetic alterations that enable growth in the absence of Snf1. However, one should expect that, if *snf1*Δ suppressor mutations arise with such a high frequency and so rapidly, they should also have been acquired in the *SNF1* knockdown mutant. This mutant was recovered on medium containing maltose as carbon source (the permissive condition), on which it nevertheless had a strong growth defect, indicating that *SNF1* was poorly expressed from the *MAL2* promoter, and this should similarly have selected for suppressor mutations. An alternative explanation for the conflicting results might therefore be that the *SNF1* knockdown mutant contains an unspecific mutation that caused the poor growth of the mutant on maltose, on which it should grow normally, and this further reduced the fitness of cells depleted for Snf1 to such an extent that no visible growth could be detected on YPD plates. Interestingly, the kinase-dead mutants generated by Vyas et al. (8) could grow on YPD plates at 37°C, like our *snf1*Δ mutants. Considering our finding that the enzymatic activity is indispensable for Snf1 function and strains containing a kinase-dead allele as the sole *SNF1* copy exhibited the same phenotypes as *snf1*Δ mutants, this would also argue that Snf1 is not essential. We conclude from our study that, under optimal conditions, C. albicans can live and grow without Snf1.

## MATERIALS AND METHODS

### Strains and growth conditions.

The C. albicans strains used in this study are listed in Table S1 in the supplemental material. All strains were stored as frozen stocks with 17.2% glycerol at −80°C and subcultured on YPD (=YP-glucose) agar plates (10 g yeast extract, 20 g peptone, 20 g glucose, 15 g agar per liter) at 30°C. Strains were routinely grown in YPD liquid medium at 30°C in a shaking incubator. For selection of transformants, 200 μg/ml nourseothricin (Werner Bioagents, Jena, Germany) or 1 mg/ml hygromycin B was added to YPD agar plates. To obtain nourseothricin-sensitive derivatives in which the *SAT1* flipper cassette was excised by FLP-mediated recombination, transformants were grown overnight in YCB-BSA-YE medium (23.4 g yeast carbon base, 4 g bovine serum albumin, 2 g yeast extract per liter, pH 4.0) without selective pressure to induce the *SAP2* promoter controlling *caFLP* expression. Appropriate dilutions were plated on YPD agar plates and grown for 2 days at 30°C. Individual colonies were picked and streaked on YPD plates as well as on YPD plates with 100 μg/ml nourseothricin to confirm nourseothricin sensitivity.

### Plasmid constructions.

The FLP-deletable *SNF1* cassette was generated in the following way (oligonucleotide primers are listed in Table S2). A fragment from the *ADH1* upstream region (positions −707 to −91) was amplified by PCR with primers AFM2-1 and AFM2-2, thereby fusing it to one copy of the *FRT* site. Similarly, a fragment containing the *FRT* site fused to a part of the *ADH1* coding sequence (positions +349 to +870) was amplified with primers AFM1-1 and AFM1-2. The *HygB* resistance marker was amplified from pGFP-HygB (19) with primers HygB-1 and HygB-2. The *SNF1* gene including 467 bp of upstream and 486 bp of downstream sequences was amplified with primers SNF1ex3-1 and SNF1ex3-2. The four fragments were combined in plasmid pSNF1ex3, such that the *SNF1* gene and the *HygB* marker are located between direct repeats of the 34-bp *FRT* site and adjacent *ADH1* sequences for genomic integration (Fig. 1B). pSAP2FL1, which contains the *ecaFLP* gene under the control of the inducible *SAP2-1* promoter, was generated by substituting the *caSAT1* selection marker for the *URA3* selection marker in the previously described plasmid pSFL213 (13). The “kinase-dead” *snf1*^K82R^ allele was generated as follows. A fragment containing the upstream region and part of the *SNF1* coding sequence was amplified with primers SNF1.12 and SNF1K82R.04; the latter changes the lysine codon AAA (positions 244 to 246 in *SNF1* allele A) into the arginine codon AGA. A fragment containing the remainder of the *SNF1* coding sequence and downstream sequences was amplified with primers SNF1K82R.03 (complementary to SNF1K82R.04) and SNF1.13. The PCR products served as the templates in a subsequent fusion PCR with primers SNF1.12 and SNF1.13. The fused fragment was then substituted for the 5′ *SNF1* flanking sequence in the *SNF1* deletion cassette contained in plasmid pSNF1M1 (2), generating pSNF1^K82R^. A *MIG1* deletion cassette was generated by amplifying the *MIG1* upstream and downstream sequences with primer pairs MIG1.01/MIG1.02 and MIG1.03/MIG1.04, respectively. The PCR products were cloned on both sides of the *SAT1* flipper cassette of pSFS5 (20) to obtain pMIG1M1. A *MIG2* deletion cassette was generated in an analogous fashion using primer pairs MIG2.01/MIG2.02 and MIG2.03/MIG2.04 to obtain pMIG2M1.

10.1128/mSphere.00805-20.2 TABLE S2 Oligonucleotide primers used in this study. Download Table S2, XLSX file, 0.01 MB. Copyright © 2020 Mottola et al. 2020 Mottola et al. This content is distributed under the terms of the Creative Commons Attribution 4.0 International license.

### Strain constructions.

C. albicans strains were transformed by electroporation (21) with the following gel-purified linear DNA fragments. The insert from plasmid pSNF1ex3 was used to integrate an FLP-deletable *SNF1* copy into the *ADH1* locus of the heterozygous *SNF1*/*snf1*Δ mutants SCSNF1M2A and -B, generating strains SCSNF1M3A and -B. The insert from plasmid pSNF1M1 was used to delete the second endogenous *SNF1* allele in strains SCSNF1M3A and -B to produce strains SCSNF1M4A and -B; removal of the *SAT1* flipper cassette from these strains yielded SCSNF1M5A and -B. The insert from plasmid pSAP2FL1 was used to integrate the *ecaFLP* gene under the control of the *SAP2-1* promoter into the *SAP2-1* allele of strains SCSNF1M5A and -B and SCSNF1M3A and -B to obtain the final conditional *snf1*Δ mutants SCSNF1M6A and -B and the control strains SCSNF1M7A and -B, respectively. Strains SCSNF1M8A and -B and SCSNF9A and -B were obtained after excision of the FLP-deletable *SNF1* copy from strains SCSNF1M6A and -B and SCSNF1M7A and -B, respectively. To generate strains that retain only the *snf1*^K82R^ allele after excision of the FLP-deletable *SNF1* copy, the insert from plasmid pSNF1^K82R^ was used to integrate the *snf1*^K82R^ allele into one of the *snf1*Δ loci of strains SNF1M5A and -B, yielding strains SNF1M10A and -B. Subsequent removal of the *SAT1* flipper cassette produced strains SNF1M11A and -B, and integration of the *ecaFLP* gene into the *SAP2-1* locus of these strains generated SMC12A and -B. Strains SCSNF1M13 A and -B are derivatives of these latter strains in which the deletable wild-type *SNF1* copy was excised by FLP-mediated recombination. The *snf4*Δ *mig1*Δ double mutants SCΔ*snf4*MIG1M4A and -B and the *snf4*Δ *mig1*Δ *mig2*Δ triple mutants SCΔ*snf4*Δ*mig1*MIG2M4A and -B were generated by sequential deletion of the *MIG1* and *MIG2* alleles in the *snf4*Δ mutants SCSNF4M4A and -B, using the inserts from plasmids pMIG1M1 and pMIG2M1.

### Isolation of genomic DNA and Southern hybridization.

Genomic DNA from C. albicans strains was isolated as described previously (17). The DNA was digested with appropriate restriction enzymes, separated on a 1% agarose gel, transferred by vacuum blotting onto a nylon membrane, and fixed by UV cross-linking. Southern hybridization with enhanced chemiluminescence (ECL)-labeled probes was performed with the Amersham ECL direct nucleic acid labeling and detection system (GE Healthcare UK Limited, Little Chalfont, Buckinghamshire, United Kingdom) according to the instructions of the manufacturer. The 5′ *SNF1* fragment from pSNF1M1, the 5′ *ADH1* fragment from pSNF1ex3, and the 5′ *SAP2* fragment from pSAP2FL1 were used as probes to analyze the structure of the corresponding genomic loci in the various strains. A probe from the *SNF1* coding region (positions +3 to +1029, amplified with primers SNF1.06 and SNF1in.04) was used to detect the presence or absence of the *SNF1* gene. A molecular size marker was included in the probes to facilitate size determination of the hybridizing genomic DNA fragments. ECL signals were captured by exposing the membranes to Hyperfilm (GE Healthcare) and digitized with an HP Scanjet 8300 (HP Inc., Palo Alto, CA).

### Growth assays.

YPD overnight cultures of the strains were adjusted to an optical density at 600 nm (OD_600_) of 2.0 in water and streaked for single colonies on YP (1% yeast extract, 2% peptone, 1.5% agar) or YNB (0.67% yeast nitrogen base with ammonium sulfate, 2% agar) medium containing 2% glucose, sucrose, or glycerol as carbon source. Growth was also tested on Spider medium (1% nutrient broth, 1% mannitol, 0.2% dipotassium phosphate, 1.35% agar). Plates were incubated for 2 days at 30°C or 37°C.

### Sequencing of *MIG1* and *MIG2*.

The *MIG1* and *MIG2* alleles in the *snf1*Δ mutants SCSNF1M8A and -B were amplified from genomic DNA with the primer pairs MIG1.01/MIG1.04 and MIG2.01/MIG2.04, respectively. Direct sequencing of the PCR products confirmed that the strains contained the wild-type *MIG2* alleles, which are identical in strain SC5314. We observed that in addition to the known single nucleotide polymorphisms, the *MIG1* alleles contained several indels that are not described in the *Candida* Genome Database (http://www.candidagenome.org/). To assign these indels to the individual *MIG1* alleles, the remaining wild-type copy in two heterozygous *mig1*Δ mutants, in which one or the other *MIG1* allele had been deleted, was amplified with primers MIG1ETfwd/MIG1ETrev and sequenced. This analysis showed that *MIG1* allele 1 is identical with C5_02940C_A, whereas *MIG1* allele 2 corresponds to C5_02940C_B but contains several additional insertions and substitutions that alter the deduced amino acid sequence of the protein in two regions. The sequence QQQQQYYQQQQQQ from positions 179–191 is changed to QQQHQQQQQYHQQQQQ, and the protein contains 12 instead of the 11 Q residues from positions 262 to 272. Manual examination of the sequence of the PCR products obtained from the *snf1*Δ mutants SCSNF1M8A and -B confirmed that the mutants had retained both original *MIG1* wild-type alleles.

### Western blotting.

Overnight cultures of the strains were diluted 10^−2^ in 50 ml fresh YPD medium and grown for 3 h at 37°C. Cells were collected by centrifugation, washed in 50 ml H_2_O, and resuspended in 500 μl breaking buffer (100 mM triethylammonium bicarbonate buffer [TEAB], 150 mM NaCl, 1% SDS, cOmplete EDTA-free protease inhibitor cocktail and PhosStop phosphatase inhibitor cocktail [Roche Diagnostics GmbH, Mannheim, Germany]) supplemented with protease and phosphatase inhibitors. An equal volume of 0.5-mm acid-washed glass beads was added to each tube. Cells were mechanically disrupted on a FastPrep-24 cell homogenizer (MP Biomedicals, Santa Ana, CA, USA) with three 40-s pulses, with 5 min on ice between each pulse. Samples were centrifuged at 13,000 rpm for 15 min at 4°C, the supernatant was collected, and the protein concentration was quantified using the Bradford protein assay. Equal amounts of protein of each sample were mixed with 1 volume of 2× Laemmli buffer, heated for 5 min at 95°C, and separated on an SDS-9% polyacrylamide gel. Separated proteins were transferred onto a nitrocellulose membrane with a mini-Protean System (Bio-Rad, Munich, Germany) and stained with Ponceau S to control for equal loading. To detect T208-phosphorylated Snf1, membranes were blocked in 5% BSA-TBST (5% bovine serum albumin–Tris-buffered saline with Tween 20) at room temperature for 1 h and subsequently incubated overnight at 4°C with phospho-AMPKα (Thr172) antibody (catalog no. 2531; Cell Signaling Technology, Danvers, MA, USA). Membranes were washed in TBST and incubated at room temperature for 1 h with anti-rabbit horseradish peroxidase (HRP) G-21234 antibody (Invitrogen GmbH, Darmstadt, Germany).
